# Uric Acid to Albumin Ratio: A Predictive Marker for Acute Kidney Injury in Isolated Tricuspid Valve Surgery

**DOI:** 10.31083/RCM26391

**Published:** 2025-02-19

**Authors:** Yaoji Liao, Liuyuan Li, Jie Li, Feifei Zhao, Chongjian Zhang

**Affiliations:** ^1^Department of Cardiac Surgery Intensive Care Unit, Guangdong Provincial People’s Hospital (Guangdong Academy of Medical Sciences), Southern Medical University, 510080 Guangzhou, Guangdong, China

**Keywords:** uric acid/albumin ratio, acute kidney injury, tricuspid valve surgery

## Abstract

**Background:**

The plasma uric acid/albumin ratio (UAR) has emerged as a novel inflammatory biomarker for predicting the development of acute kidney injury (AKI) following percutaneous coronary intervention. However, the potential of the UAR to serve as a predictive marker for AKI in patients undergoing isolated tricuspid valve (TV) surgery remains unknown. This study aimed to explore the association between the UAR and AKI and to assess whether the UAR can predict AKI in these patients.

**Methods:**

We conducted a retrospective analysis of patients who underwent isolated TV surgery between January 2018 and June 2019. The patients were divided into three groups based on the tertiles of the UAR. We utilized multivariate logistic regression and restricted cubic spline analysis to examine the association between the UAR and AKI. Additionally, we used the receiver operating characteristic (ROC) curve analysis to assess the predictive accuracy of the UAR for AKI.

**Results:**

A total of 224 patients were enrolled in this study, of whom 41 developed AKI. The incidence of AKI across the three UAR tertiles was 3.8%, 22.2%, and 29.7%, with a significant difference between the group (*p* < 0.001). In the multivariate analysis, UAR ≥8.5 was associated with a 7-fold increased risk of AKI (odds ratio (OR): 7.73, 95% confidence interval (CI): 1.61–37.14), while a UAR ≥10.8 was a linked to a 9-fold increased risk (OR: 9.34, 95% CI: 1.96–44.60). The restricted cubic spline model showed a linear association between the UAR and AKI development. The area under the curve (AUC) value for the UAR was 0.713 (95% CI: 0.633–0.793; *p* < 0.001) with a cutoff value of 8.89.

**Conclusions:**

An increased UAR was significantly associated with a higher risk of AKI in patients undergoing isolated TV surgery; however, while the UAR could serve as a marker to predict AKI, it was not superior to uric acid alone.

## 1. Introduction 

Acute kidney injury (AKI) is a common significant complication following cardiac surgery [1]. The development of AKI is associated with prolonged hospital stays, an increased need for renal replacement therapy, and higher mortality rates both in the short and long term [2]. The incidence of postoperative AKI in patients receiving tricuspid valve (TV) surgery and cardiac valve replacement is estimated to be 30% and 46.8%, respectively [3, 4]. Patients demonstrated a 1.58-fold increased risk of AKI after tricuspid valve replacement [3]. Although some novel biomarkers, such as urinary liver fatty acid binding protein (L-FABP), urinary neutrophil gelatinase-associated lipocalin (NGAL), and kidney injury molecule 1 (KIM-1) have been studied for AKI stratification and prediction [5], the cost-effectiveness of using these in clinical practice remains unclear [6]. Therefore, predicting AKI with readily calculable markers before tricuspid valve surgery is essential for identifying high-risk patients and implementing preventative strategies.

Inflammation and oxidative stress are key pathophysiologies in cardiac surgery-related AKI [7]. Uric acid enhances proinflammatory mediators, leading to glomerulosclerosis and tubular damage [8]. The redox state of serum albumin, indicative of systemic antioxidant capacity, is associated with reduced renal function [9]. Both serum uric acid and albumin serve as an independent risk marker for AKI [10, 11]. However, conditions such as malnutrition, sepsis, inflammation, congestive heart failure, and chronic kidney disease can influence their levels [12, 13, 14]. The serum uric acid/albumin ratio (UAR), comprising serum uric acid and serum albumin levels, has emerged as a novel inflammatory biomarker for predicting the onset of AKI [14] and is associated with short-term mortality in patients with acute kidney injury [15]. This marker has been evaluated for its predictive value in post-contrast acute kidney injury following percutaneous coronary intervention (PCI) [16]. However, the clinical features and underlying pathophysiological processes differ between contrast-induced acute kidney injury after PCI and acute kidney injury associated with cardiac surgery. There is a notable absence of research validating the predictive value of this marker in patients following tricuspid valve surgery. Consequently, this study aimed to determine whether the UAR can predict the risk of AKI in those who have undergone such surgery.

## 2. Materials and Methods

### 2.1 Study Design and Population

**Fig. 1.**
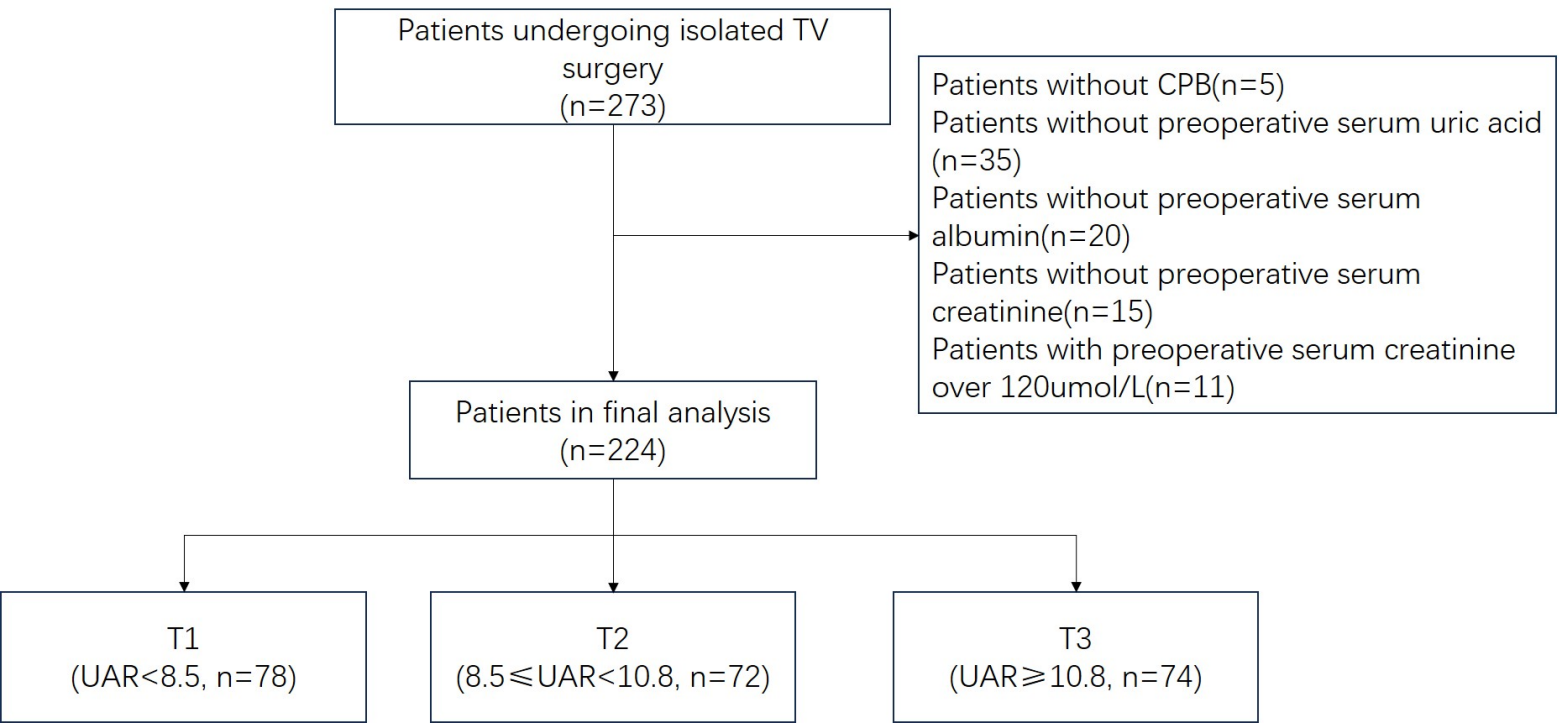
**Schematic chart of enrolled patients**. TV, tricuspid valve; CPB, cardiopulmonary bypass; UAR, uricacid/albumin ratio.

This study included 224 patients and was conducted in the thirty-bed cardiac surgery intensive care unit (ICU) at Guangdong Provincial People’s Hospital in Guangzhou, China, from January 2018 to June 2019. Eligible participants were 18 years of age or older and had undergone isolated tricuspid valve repair or replacement surgery with cardiopulmonary bypass support. Initially, the study had 273 participants, but 49 were excluded due to the lack of cardiopulmonary bypass support, preoperative laboratory data, or serum creatinine levels exceeding 120 µmol/L (Fig. 1). After ranking the UAR values from the lowest to the highest, the UAR values at the 1/3 and 2/3 quantiles were established as the cutoff points for stratifying patients into low, medium, and high UAR groups. These patients were then stratified again into three tertiles based on their UAR levels: UAR <8.5, 8.5 ≤ UAR < 10.8, and UAR ≥10.8. This research protocol was approved by the Ethics Committee of Guangdong Provincial People’s Hospital (KY2024-605–02).

### 2.2 Data Collection

Baseline demographic and clinical data, including age, sex, height, weight, and co-morbidities, were extracted from the hospital’s electronic medical records system. Preoperative cardiac function, specifically left ventricular ejection fraction (LVEF) and pulmonary artery pressure, was assessed using the latest echocardiography. Additionally, preoperative laboratory parameters such as creatinine, serum uric acid, albumin, hemoglobin, blood glucose levels, and platelet counts were documented before surgery. Intraoperative data were also recorded, including cardiopulmonary bypass (CPB) circulation time and aortic cross-clamp time. Postoperative outcomes were monitored, specifically the incidence of AKI, mechanical ventilation duration, and ICU stay length. The UAR was calculated as the ratio of serum uric acid to serum albumin. AKI was defined according to the Kidney Disease Improving Global Outcomes (KDIGO) criteria [17] and was assessed by a trained physician in the ICU.

### 2.3 Statistical Analysis

Continuous variables are described as the mean ± standard deviation (SD) or median interquartile range (IQR). The Kruskal–Wallis H test was employed to compare variables among the three groups, as the Kolmogorov–Smirnov test determined the distribution to be non-normal. Categorical variables were expressed as frequency and percentages, and their intergroup comparisons were conducted using the Chi-square or Fisher’s exact test. Univariate logistic regression was performed to evaluate the risk factors associated with the development of AKI. A multivariate logistic regression model assessed the relationship between the UAR and AKI development. Uric acid and albumin were not included in the multivariable model with the UAR to avoid issues with multicollinearity and interaction. Model I adjusted the covariates for sex, height, weight, age, hypertension, diabetes, and atrial fibrillation. Model II included additional covariates: hemoglobin, platelets, blood glucose, creatinine, LVEF, pulmonary artery pressure, blood transfusion, CPB duration, and cross-clamp time. Restricted cubic spline analysis was used to explore the non-linear relationship between the UAR and the incidence of acute kidney injury among subjects undergoing isolated TV surgery. The predictive values of the UAR, serum uric acid, and albumin levels for AKI were evaluated using receiver operating characteristic (ROC) curve analysis, and a statistical comparison was made using the DeLong test. All statistical analyses were performed using R version 4.2.3 in RStudio (2024.09.1+394, Posit, Boston, MA, USA), with *p*-values < 0.05 considered statistically significant.

## 3. Results

### 3.1 Baseline Characteristics

The clinical characteristics of patients are presented in Table 1. A total of 224 patients participated in the analysis; 223 patients underwent tricuspid valve repair, and one patient underwent tricuspid valve replacement. Patients were further divided into three tertiles, and the median (IQR) of the UAR levels were 7.4 (6.5–8.0) in T1 (n = 78), 9.5 (9.0–10.1) in T2 (n = 72), and 12.7 (11.7–14.4) in T3 (n = 74). Sex, age, weight, UAR, uric acid, albumin, creatinine, CPB duration, and blood transfusion significantly differed among the three groups. Patients in the T3 group were predominantly males with higher age, weight, uric acid, creatinine, and lower albumin. As the UAR tertile increased, patients experienced longer durations of mechanical ventilation and ICU stay, yet there were no differences in the in-hospital mortality rates among the groups. A total of 41 out of 224 (18.3%) patients developed AKI. The incidence of AKI increased significantly across the increasing UAR tertiles, with rates of 3.8%, 22.2%, and 29.7% in each successive tertile, respectively (*p* < 0.001).

**Table 1.** **Baseline characteristics of patients stratified by the UAR tertile**.

Variables		T1 (n = 78)	T2 (n = 72)	T3 (n = 74)	*p*-value
		UAR <8.5	8.5 ≤ UAR < 10.8	UAR ≥10.8	
Male, n (%)					0.006
	No	62 (79.5)	50 (69.4)	41 (55.4)	
	Yes	16 (20.5)	22 (30.6)	33 (44.6)	
Age, years		40.0 [28.0; 51.0]	40.0 [30.5; 54.0]	48.5 [36.0; 58.0]	0.01
Height, cm		159.2 ± 6.3	159.4 ± 8.9	161.3 ± 8.6	0.2
Weight, kg		52.5 [47.0; 60.0]	54.9 [50.0; 62.0]	58.2 [51.5; 66.0]	0.016
Hypertension, n (%)					0.626
	No	73 (93.6)	68 (94.4)	67 (90.5)	
	Yes	5 (6.4)	4 (5.6)	7 (9.5)	
Diabetes, n (%)					0.155
	No	77 (98.7)	67 (93.1)	72 (97.3)	
	Yes	1 (1.3)	5 (6.9)	2 (2.7)	
Atrial fibrillation , n (%)					0.078
	No	67 (85.9)	59 (81.9)	53 (71.6)	
	Yes	11 (14.1)	13 (18.1)	21 (28.4)	
LVEF, %		64.0 [61.0; 68.0]	63.0 [60.0; 67.0]	64.0 [60.0; 69.0]	0.254
Pulmonary artery pressure, mmHg		45.5 [35.0; 62.0]	40.5 [32.0; 54.0]	45.0 [35.0; 56.0]	0.203
Uric acid/albumin ratio		7.4 [6.5; 8.0]	9.5 [9.0; 10.1]	12.7 [11.7; 14.4]	<0.001
Uric acid, umol/L		297.9 [264.2; 332.0]	384.5 [362.0; 419.3]	494.0 [434.0; 554.0]	<0.001
Albumin, g/L		40.8 [38.8; 42.5]	40.4 [38.3; 42.4]	38.0 [35.8; 40.3]	<0.001
Hemoglobin, g/L		131.0 [117.0; 140.0]	132.5 [122.0; 147.0]	136.5 [121.0; 147.0]	0.132
Platelets, 10^9^/L		225.5 [186.0; 278.0]	223.0 [182.0; 254.0]	202.5 [161.0; 251.0]	0.069
Blood glucose, mmol/L		4.6 [4.2; 5.0]	4.4 [4.2; 4.9]	4.5 [4.2; 4.9]	0.923
Creatinine, mg/dL		59.5 [53.0; 67.9]	66.3 [57.0; 74.4]	73.5 [61.9; 84.6]	<0.001
CPB duration, minutes		101.0 [81.0; 128.0]	102.0 [80.0; 138.0]	124.0 [92.0; 148.0]	0.022
Cross-clamp time, minutes		48.0 [35.0; 70.0]	40.5 [26.5; 64.0]	41.0 [0.0; 75.0]	0.235
Blood transfusion, n (%)					0.048
	No	68 (87.2)	51 (70.8)	58 (78.4)	
	Yes	10 (12.8)	21 (29.2)	16 (21.6)	
Acute kidney injury, n (%)					<0.001
	No	75 (96.2)	56 (77.8)	52 (70.3)	
	Yes	3 ( 3.8)	16 (22.2)	22 (29.7)	
Duration of mechanical ventilation, hours		9.0 [5.0; 17.0]	9.5 [5.0; 19.5]	16.0 [6.0; 23.0]	0.035
Length of stay in ICU, hours		39.0 [20.0; 46.0]	41.5 [22.0; 67.0]	47.0 [39.0; 86.0]	<0.001
In-hospital death					0.119
	No	78 (100.0)	70 (97.2)	74 (100.0)	
	Yes	0 (0.0)	2 (2.8)	0 (0.0)	

Abbreviations: LVEF, left ventricular ejection fraction; CPB, cardiopulmonary bypass; ICU, intensive care unit; UAR, uric acid/albumin ratio.

### 3.2 Association of UAR and Acute Kidney Injury Development

Potential factors associated with AKI development are shown in Table 2. Sex, height, age, hypertension, atrial fibrillation, creatinine, platelet count, blood transfusion, and UAR were significantly associated with AKI development. Compared to the lowest tertile (T1), the middle (T2) and the highest (T3) tertiles of the UAR were associated with a seven-fold (odds ratio (OR): 7.14, 95% confidence interval (CI): 1.99–25.7) and ten-fold (OR: 10.58, 95% CI: 3.01–37.17) increased risk of AKI following TV surgery, respectively.

**Table 2.** **Univariate logistic regression analysis for risk factors of AKI development**.

Variables		Acute kidney injury		
		OR	95% CI	*p*-value
UAR tertile				
	T1	reference		
	T2	7.14	1.99–25.70	0.003
	T3	10.58	3.01–37.17	<0.001
Male, %		3.59	1.79–7.23	<0.001
Height, cm		1.05	1.00–1.09	0.037
Weight, kg		1.01	0.98–1.05	0.41
Age, years		1.04	1.01–1.07	0.002
Hypertension, %		2.97	1.01–8.69	0.048
Diabetes, %		2.81	0.64–12.27	7.169
Atrial fibrillation, %		3.4	1.62–7.14	0.001
Hemoglobin, g/L		1	0.98–1.01	0.57
Platelets, 10^9^/L		0.99	0.98–0.99	<0.001
Blood glucose, mmol/L		0.93	0.62–1.40	0.735
Creatinine, mg/dL		1.03	1.00–1.05	0.018
LVEF		0.99	0.97–1.02	0.636
Pulmonary artery pressure, mmHg		0.98	0.97–1.00	0.102
Blood transfusion, %		2.72	1.30–5.71	0.008
CPB duration time, minutes		1.01	1.00–1.01	0.052
Cross-clamp time, minutes		0.99	0.98–1.00	0.146

Abbreviations: UAR, uric acid/albumin ratio; OR, odds ratio; CI, confidence interval; LVEF, left ventricular ejection fraction; CPB, cardiopulmonary bypass; AKI, acute kidney injury.

**Fig. 2.**
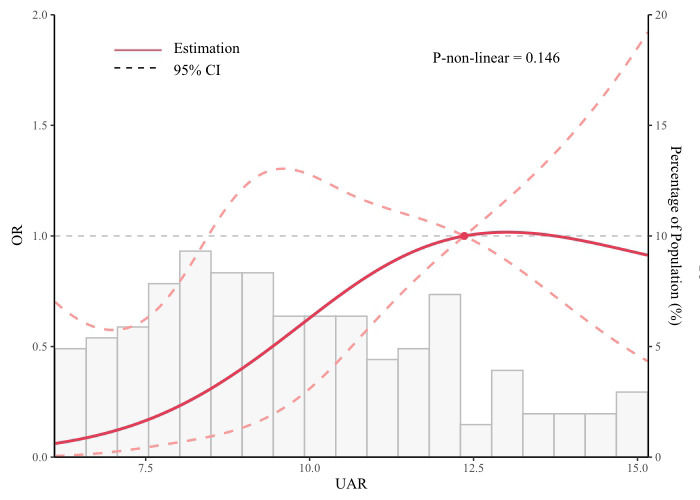
**Restricted cubic spline curves for AKI by TV surgery**. OR, odds ratio; CI, confidence interval; UAR, uric acid/albumin ratio; AKI, acute kidney injury; TV, tricuspid valve.

Table 3 presents the results of multivariate logistic regression analyses assessing the association between UAR and the development of AKI in both the unadjusted and adjusted models. When the UAR was used as a continuous variable, the unadjusted model and Models I and II adjusted for confounding variables demonstrated an accelerated risk of AKI by 1.69, 1.56, and 1.7 times, respectively, for each standard deviation increase in the UAR level. The UAR was categorized into three levels, with the lowest tertile used as the reference group. In the unadjusted model, both the middle and the highest tertiles demonstrated a statistically significant increase in the risk of developing AKI, with ORs of 7.14 (95% CI: 1.99–25.7; *p* = 0.003) and 10.58 (95% CI: 3.01–37.17; *p* < 0.001), respectively. After adjusting for demographic and clinical variables in Model I, the association between the higher UAR tertiles and AKI risk remained robust, with ORs of 8.75 (95% CI: 2.18–35.13; *p* = 0.002) for the middle tertile and 9.28 (95% CI: 2.43–35.41; *p* = 0.01) for the highest tertile. Further adjustment for laboratory results, LVEF, pulmonary pressure, CPB duration, and cross-clamp time in Model II did not attenuate the association. The ORs for the middle tertile and the highest tertile were 7.73 (95% CI: 1.61–37.14; *p* = 0.011) and 9.34 (95% CI: 1.96–44.60; *p* = 0.005), respectively, indicating a persistent and significant increase in AKI risk with higher UAR levels. A significant positive trend was observed in the development of AKI across increasing UAR tertile levels in both the unadjusted and adjusted models, with all *p*-values for the trend indicating significance (*p* < 0.05). Meanwhile, the restricted cubic spline model also showed a linear association between the UAR and AKI development (non-linear *p* = 0.146) (Fig. 2).

**Table 3.** **Multivariate logistic regression analysis of the relationship between UAR and AKI development**.

		Crude model			Model I			Model II		
		OR	95% CI	*p*-value	OR	95% CI	*p*-value	OR	95% CI	*p*-value
UAR, per 1SD increased		1.69	1.22–2.34	0.001	1.56	1.11–2.20	0.011	1.7	1.06–2.74	0.027
UAR tertile										
	T1	1 (Ref)			1 (Ref)			1 (Ref)		
	T2	7.14	1.99–25.70	0.003	8.75	2.18–35.13	0.002	7.7	1.61–37.14	0.011
	T3	10.58	3.01–37.17	0.001	9.28	2.43–35.41	0.001	9.3	1.96–44.60	0.005
*p*-value for the trend		<0.001			0.002			0.013		

Crude model: unadjusted; Model I: adjusted for male, height, weight, age, hypertension, diabetes, and atrial fibrillation; Model II: adjusted for Model I and hemoglobin, platelets, blood glucose, creatinine, LVEF, pulmonary artery pressure, blood transfusion, CPB duration time, and cross-clamp time. UAR, uric acid/albumin ratio; OR, odds ratio; CI, confidence interval; SD, standard deviation; LVEF, left ventricular ejection fraction; CPB, cardiopulmonary bypass; AKI, acute kidney injury.

### 3.3 Predictive Value of the UAR for AKI Development

**Fig. 3.**
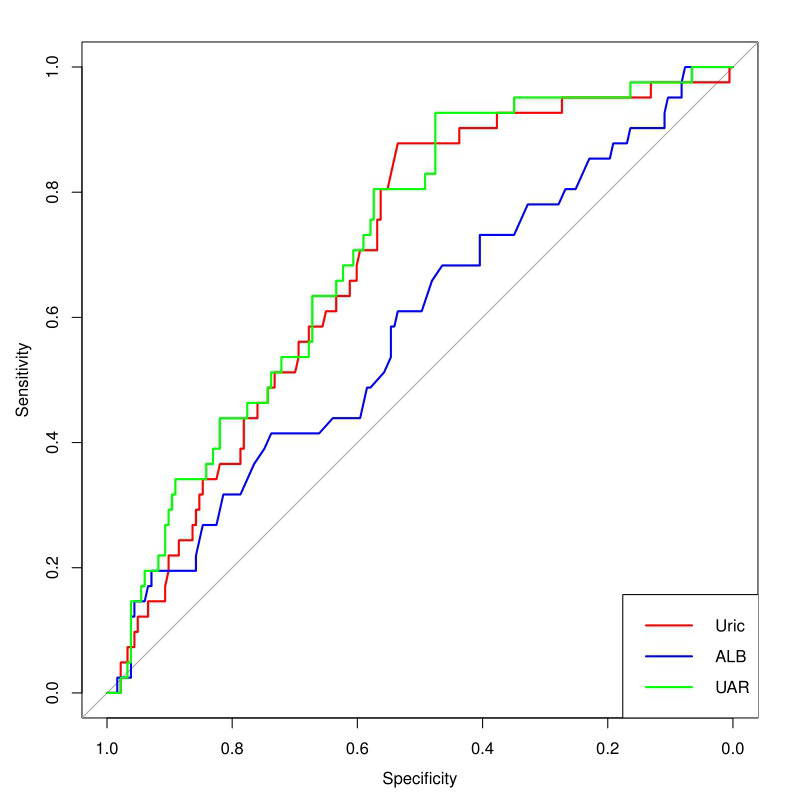
**Receiver operating characteristic curve analysis of the UAR, uric acid, and albumin levels to predict AKI**. Uric, uric acid; ALB, albumin; UAR, uric acid/albumin ratio; AKI, acute kidney injury.

ROC analysis presented in Fig. 3, the area under the curve (AUC) of the UAR was 0.713 (95% CI: 0.633–0.793; *p* < 0.001) with a cutoff value >8.89; the AUC for uric acid was 0.695 (95% CI: 0.613–0.777; *p* < 0.001). However, albumin demonstrated a weaker predictive value with an AUC of 0.583 (95% CI: 0.485–0.682) and a non-significant *p*-value of 0.952. The DeLong test showed that the UAR is a superior predictor of AKI compared to albumin, with a z-score of 2.35 (95% CI: 0.02–0.23; *p* = 0.01). However, the UAR did not show superiority over uric acid as a predictor, with a z-score of 0.95 (95% CI: –0.01–0.19; *p* = 0.34).

## 4. Discussion

Our study confirms that the UAR can be used as an independent predictor of AKI in patients undergoing isolated tricuspid valve repair or replacement surgery. A linear relationship between the UAR levels and AKI incidence suggests that an elevated UAR is linked to an increased risk of AKI, even after accounting for confounding factors. The UAR has a moderate predictive value for AKI, with its predictive efficacy equivalent to uric acid.

Our study found that individuals in the higher UAR tertile were older, predominantly male, and had elevated levels of creatinine and uric acid and lower levels of albumin. These findings are consistent with previous studies [15, 18]. For example, the study by Li *et al*. [18] observed that patients with unstable angina pectoris who underwent percutaneous coronary intervention and had a high UAR of ≥8.38 and exhibited a higher all-cause mortality rate during follow-up outside the hospital compared to those with a UAR <8.38. Similarly, the study by Özgür *et al*. [15] reported that patients with a UAR greater than 2.36 had an increased 30-day mortality rate compared to those with a UAR below 2.36. However, our study did not find a correlation between the UAR and ICU mortality. There are a couple of reasons for this discrepancy. Firstly, an international cross-study by Hoste* et al*. [19] reported that the mortality rate for patients with AKI in the ICU is approximately 24%. Given this benchmark, the relatively low mortality rate observed in our study may not be sufficient to demonstrate a statistically significant difference. Secondly, this discrepancy could suggest that the UAR is more strongly associated with long-term mortality outcomes than short-term mortality in the ICU.

Acute kidney injury is characterized by a sudden decline in renal function within hours to days, indicated by a rapid rise in serum creatinine levels, a decrease in urine output, or both [7]. The incidence of AKI related to cardiac surgery is estimated to be between 20% and 30% [2, 7, 20, 21]. In this study, 18.3% of patients developed AKI following surgery. Several risk factors, including age, male gender, hypertension, cardiac arrhythmia, transfusion, and preoperative serum creatinine levels, have been associated with the development of AKI following cardiac surgery [21, 22, 23]. Our study corroborates these associations, demonstrating that older male patients with higher creatinine levels, lower platelet counts, history of transfusion, hypertension, and atrial fibrillation are at an increased risk of AKI development. Our study determined that the UAR is a risk factor for AKI, with a linear relationship indicating that higher UAR levels are associated with an increased risk of AKI. Furthermore, elevated uric acid and decreased albumin levels may contribute to this condition. Serum uric acid has been widely evaluated as a predictive biomarker for AKI after cardiac surgery. A study of 190 patients undergoing coronary artery bypass surgery revealed that for each 1-unit increase in serum uric acid concentration, the risk of AKI rose by 1.18 times [24]. Another study involving 247 cardiac surgery patients showed that, after adjusting for confounders with multivariate logistic regression, a preoperative uric acid level of ≥373 µmol/L was linked to a 5.4-fold increase in AKI risk compared to levels ≤373 µmol/L [25]. However, a study conducted by Lapsia *et al*. [26] analyzed the incidence of AKI and all available uric acid values in 4949 adult patients who underwent cardiovascular surgery, starting from the lowest value with incremental increases of 0.5 mg/dL, and found that a J-shaped relationship appears to exist between uric acid (UA) and AKI. These data suggest that both hypouricemia and hyperuricemia are linked to an increased risk of acute kidney injury; however, the study did not specify the predictive threshold for uric acid levels. Thus, detecting a new biomarker or considering their combination may be needed for AKI prediction. Lower serum albumin is an independent factor for both the development of AKI and post-AKI mortality [10]. In a study of 634 diabetes mellitus patients undergoing coronary bypass grafting, low preoperative serum albumin levels predicted AKI independently, with an albumin level below 3 mg/dL showing an AUC of 0.621 for AKI prediction (*p* < 0.001) [27]. Furthermore, a separate study of 1182 patients undergoing off-pump coronary artery bypass surgery found that preoperative serum albumin levels below 4.0 g/dL were associated with a 1.83-fold higher risk of AKI than levels above this threshold [28]. Elevated uric acid and reduced albumin levels indicate an increased UAR, which is associated with a higher risk of AKI.

Studies by Şaylık *et al*. [29] and Yeter *et al*. [14] have demonstrated the predictive value of the UAR for kidney impairment post-percutaneous coronary intervention. Şaylık *et al*. [29] identified a UAR cutoff of >1.62 for detecting contrast-induced nephropathy (CIN), while Yeter *et al*. [14] found that a UAR >1.7 correlates with AKI in critically ill patients. In our study, a UAR value exceeding 8.89 was the optimal threshold for AKI detection in patients undergoing TV surgery. The discrepancy in our study may stem from different units for uric acid and albumin: µmol/L for UA and g/L for albumin (ALB), contrasting with mg/dL for UA and g/dL for ALB in their studies. In our study, the UAR demonstrated a moderate predictive value for AKI following TV surgery. Comparative analysis of the ROC curves revealed that the predictive accuracy of the UAR did not exceed that of serum uric acid. This outcome contrasts previous studies suggesting UAR as a superior predictor for post-contrast kidney injury or nephropathy in PCI [16, 29]. The differences in albumin levels can explain this. In contrast to the study by Zhang *et al*. [16], which documented the lowest albumin level at 33.3 ± 0.45 g/L, our study reported a median level of 38 g/L, with a 95% CI ranging from 35.8 to 40.3 g/L. A previous study explored the relationship between serum albumin and AKI in 624 patients undergoing surgery for acute type A aortic dissection and noted that the risk of AKI does not significantly increase once albumin levels exceed a threshold of 32 g/L [30]. Furthermore, the AUC for albumin in predicting AKI in our study was 0.583, with a *p*-value of 0.952, suggesting a poor predictive value. Consequently, the predictive efficacy of the UAR appears to be more reliant on uric acid levels than albumin levels. Therefore, our study found the predictive accuracy of the UAR and uric acid level to be quite comparable.

The metabolic disorder of uric acid and decreased albumin synthesis before cardiac surgery may underlie the mechanism through which the UAR predicts AKI. Firstly, the kidney plays a significant role in uric acid excretion and absorption. Therefore, before undergoing cardiac surgery, most patients grapple with diminished cardiac function, potentially triggering a cascade of events known as cardiorenal syndrome, which in turn can impair kidney function [31]. Consequently, this leads to reduced excretion of uric acid in the urine and increased reabsorption, resulting in elevated uric acid levels in the bloodstream [32]. Secondly, sustained elevation in uric acid levels may promote pathogenic inflammation, cellular proliferation, and maladaptive changes. These pathological changes can result in glomerulosclerosis and fibrosis in the tubulointerstitium, resulting in renal damage even after cardiac surgery [33]. Chronic kidney disease is characterized by persistent chronic inflammation, which may decrease albumin concentration due to a reduced synthesis rate [31, 34]. As a result, low serum albumin levels indicate the severity of the inflammation [35] and reflect the extent of kidney function impairment.

The UAR has a wide range of applications. Moreover, it is effective in predicting AKI and has demonstrated significant prognostic value in forecasting outcomes for patients with ST-elevation myocardial infarction (STEMI) [36] and complications following various procedures, including cryoballoon catheter ablation [37], coronary artery bypass grafting (CABG) [38], and PCI. Compared with single markers, the UAR provides stronger results and a multidimensional impact, making it particularly advantageous in clinical practice.

## 5. Limitations

The current study has several inherent limitations. Firstly, the retrospective design of the study prevents us from establishing a causality relationship between the UAR and AKI. Secondly, the study focuses solely on patients who underwent isolated TV surgery, which may limit the generalizability of our findings to other cardiac surgery populations. Additionally, the absence of long-term follow-up data limits our understanding of AKI incidence over an extended period. Finally, despite applying a multivariate analysis, residual confounders, such as the duration of tricuspid valve disease, right heart failure, and diuretic therapy, could not be fully addressed. For these reasons, future research should be prospective and include diverse patient populations to validate our findings. We strongly recommend incorporating long-term follow-up data to gain a deeper understanding of the incidence and progression of AKI.

## 6. Conclusions

Our analysis establishes a clear linear correlation between preoperative UAR levels and the incidence of AKI in patients undergoing isolated tricuspid valve repair or replacement. An elevated UAR is linked to an increased risk of AKI. The UAR demonstrates a moderate level of predictive accuracy for AKI; while it is comparable to uric acid alone, it has wider clinical application value. As a cost-effective biomarker, UAR could be instrumental in identifying patients at risk of AKI who may require closer monitoring and management.

## Availability of Data and Materials

All data relevant to the study are included in the article. Data can also be requested from the corresponding author.
